# Inactivating UBE2M Impacts the DNA Damage Response and Genome Integrity Involving Multiple Cullin Ligases

**DOI:** 10.1371/journal.pone.0101844

**Published:** 2014-07-15

**Authors:** Scott Cukras, Nicholas Morffy, Takbum Ohn, Younghoon Kee

**Affiliations:** 1 Department of Cell Biology, Microbiology, and Molecular Biology, College of Arts and Sciences, University of South Florida, Tampa, Florida, United States of America; 2 Department of Cellular & Molecular Medicine, College of Medicine, Chosun University, Gwangju, Republic of Korea; Universita' di Milano, Italy

## Abstract

Protein neddylation is involved in a wide variety of cellular processes. Here we show that the DNA damage response is perturbed in cells inactivated with an E2 Nedd8 conjugating enzyme UBE2M, measured by RAD51 foci formation kinetics and cell based DNA repair assays. UBE2M knockdown increases DNA breakages and cellular sensitivity to DNA damaging agents, further suggesting heightened genomic instability and defective DNA repair activity. Investigating the downstream Cullin targets of UBE2M revealed that silencing of Cullin 1, 2, and 4 ligases incurred significant DNA damage. In particular, UBE2M knockdown, or defective neddylation of Cullin 2, leads to a blockade in the G1 to S progression and is associated with delayed S-phase dependent DNA damage response. Cullin 4 inactivation leads to an aberrantly high DNA damage response that is associated with increased DNA breakages and sensitivity of cells to DNA damaging agents, suggesting a DNA repair defect is associated. siRNA interrogation of key Cullin substrates show that CDT1, p21, and Claspin are involved in elevated DNA damage in the UBE2M knockdown cells. Therefore, UBE2M is required to maintain genome integrity by activating multiple Cullin ligases throughout the cell cycle.

## Introduction

Protein neddylation (Nedd8 conjugation) is involved in a wide variety of cellular processes. E1 Nedd8 activating enzyme is a heterodimer of UBA3 and NAE1, which function with the two known E2 conjugating enzymes UBE2M and UBE2F [1]. The E2 enzymes promote neddylation of several known targets, including the Cullin components of the CRL (Cullin Ring Ligase) complexes, p53, and histone H4 [1]–[4]. Conjugation of Nedd8 onto the Cullin subunits leads to activation of the ubiquitin ligase activity [4], [5]. UBE2M interacts with the RBX1 component of CRL complexes, thereby promoting neddylation of Cullin (CUL) 1, 2, 3, and 4, whereas UBE2F interacts with RBX2, which promotes neddylation of CUL5 [1]. Individual CRL E3 complexes can associate numerous adaptor subunits that provide substrate specificity; CUL1 associates with F-Box proteins, CUL2 ligase associates with VHL box proteins, CUL3 associates with BTB3-containing proteins, and CUL4 associates with DCAFs (DDB1-CUL4 Associated factor) [6]–[10]. In addition to RBX1 and RBX2, RNF111 serves as an E3 component in the neddylation system that promotes histone neddylation in conjunction with UBE2M [2]. DNA damage response (DDR) and cell cycle checkpoint controls are among the diverse pathways that are regulated by Cullins [11]–[13]. To name a few mechanisms, CUL1 forms a complex with a F-box protein β-TRCP to regulate degradation of several cell cycle checkpoint and DDR proteins, including CDC25A, WEE1, CLASPIN, FANCM, and MDM2 [14]–[20]. CUL4-DDB2 complex induces degradation of nucleotide excision repair factor XPC [21] and also ubiquitinate Histones to facilitate DDR [22], and CUL4-CDT2 complex controls replication by degrading CDT1, p21, and SET8 [23]–[30].

Development of an investigational pharmacological inhibitor (MLN4924) of the NAE1 E1 component provided a proof of principle that inactivating the neddylating enzyme can be an effective approach for targeting cancer cells [31]. Treatment of MLN4924 in cultured cells leads to DNA damage, checkpoint activation, cellular senescence and apoptosis, and suppression of tumor growth in a mice model [31], [32]. Induction of DNA re-replication and p21-mediated cell cycle arrest has been primarily attributed to growth suppression [33], [34]. Suppressing the overall neddylation affects cellular response to conventional DNA damaging agents, shown by increased sensitivity of cancer cells to DNA damaging agents [33], [35]–[38]. Disrupting the normal DNA damage response has been proposed as a module for increasing drug sensitivity in cancer cells. For instance, targeting the proteasome or CDK1 has been shown to compromise normal DNA repair activity and cellular response to DNA damaging agents [39]–[41].

Here we investigated the effects of inhibiting the E2 neddylating enzyme UBE2M on the overall DNA damage response. Given the primary role of UBE2M in neddylating Cullins, we comprehensively analyzed the effects of ablating individual Cullins in genome integrity. We show that multiple Cullin ligases impact different aspects of DNA damage response and genome integrity. These data provide mechanistic information for the effects of inhibiting protein neddylation on genomic integrity, and support the notion that inhibiting the E1/E2 neddylating enzymes or individual Cullins can be exploited for disrupting normal cellular response to DNA damaging agents.

## Experimental Procedures

### Cell lines, plasmids, and chemicals

HeLa, 293T, and U2OS cells were grown in Dulbecco's Modified Eagle's Medium (DMEM) supplemented with 10% Bovine serum and L-glutamine. HEY ovarian cancer cells (gift from Dr. Meera Nanjundan [42]; STR profiled) and HCT116 cells (WT and p21-/-; gift from Dr. Bert Vogelstein) were grown in RPMI, or McCoy's Medium supplemented with 10% Bovine serum and L-glutamine. These cells were all mycoplasma tested. All cells were grown in 37°C in 5% CO_2_. UBE2M cDNA was cloned to pOZ-N retroviral vector for expression studies. Site-directed mutagenesis for UBE2M C111S and CUL2 K689R were conducted following the QuikChange Site-Directed Mutagenesis protocol by Stratagene. pOZ-FLAG-HA-UBE2M and pcDNA-Myc3-CUL2 plasmids were used as templates, respectively. MLN4924 was purchased from ActiveBiochem. PARP inhibitor (ABT-888) and Camptothecin were purchased from Selleck chemicals.

### RNAi

Cells were cultured in medium without antibiotics and transfected once with 20 nM siRNA using RNAiMAX (Invitrogen) reagent following the manufacturer's protocol.

The following siRNA sequences were used: CUL1: 5′-AUUCCAGGCCAACAAACUGAGCUCC-3′

CUL2 #1: 5′-GCCCUUACGUCAGUUGUAAAUUACA-3′, CUL2 #2: 5′-UUAGCAAGCAGUUCAGGUGCUUUGC-3′

CUL2 #3: 5′- AACCUAAUAAUUGUAUCUACA-3′

UBE2F #1: 5′-CGGAGGGUUUCUGUGAGAGACAAAU-3′,

UBE2F #2: 5′- UGAUGUAGUCAUCCACUUUAUUCCG-3′,

CDT1 #1: 5′-CCGCGCUUCAACGUGGAUGAA-3′

CDT1 #2: 5′-CACCUGGUGGAUUCACAUUAA-3′

p21: 5′-AAGACCAUGUGGACCUGUCAC-3′, CLASPIN: 5′-GACGCGAAGCAUCUUCCAAAUA-3′

IKB-α: 5′-AAGGGUGUACUUAUAUCCACA-3′, IKB-β: 5′-CACGUGGCCGUUAUCCACAAA-3′

WEE1: 5′-CACUGGUAAAGCAUUCAGUAU-3′, NFR2: 5′-AAGGATTATGACTGTTAA-3′, BRCA1: 5′- CAGCAGTTTATTACTCACTAA-3′

CUL4A: 5′-AAAUGAAUCUUUAUACACCUGCAGG-3′,

UBE2M #1: 5′-GGGCUUCUACAAGAGUGGGAAGUUU-3′,

UBE2M #2: 5′-ACUCCAUAAUUUAUGGCCUGCAGUA-3′,

CDT2 #1: 5′-CCGAGUCUACUGGGUAUAACA-3′

CDT2 #2: 5′-CUGGGAUACCAGGUGCAACAA-3′

pLKO-vector based lenti-viral shRNA vectors were purchased from Sigma Aldrich. The lentiviral vectors were transfected into 293T packaging cells with helper plasmids. 48 hrs after transfection, virus-containing supernatant was harvested, filtered, then used for infecting recipient cells (e.g. HEY, HeLa). The sequences of shRNA are following:

CUL4A: CCGGACTGTTTAGAACCCATATTATCTCGAGATAATATGGGTTCTAAACAGTTTTTTG

UBE2M: CCGGCGATGGGAAATGAATTGGCTTCTCGAGAAGCCAATTCATTTCCCATCGTTTTT

### Western blots

Cell extracts were run on an SDS-PAGE gel and then transferred to a PVDF membrane (Bio-Rad, Hercules, CA). Membranes were probed with primary antibodies overnight at 4°C. The membranes were then washed and incubated with either mouse or rabbit secondary antibody linked with horseradish peroxidase (Cell Signaling Technologies) and washed. The bound antibodies were viewed via Pierce ECL Western Blotting Substrate (Thermo Scientific). The following primary antibodies were used: α-CUL1, α-CUL3 α-CUL4A, α-CHK1, α-CLASPIN, α-p21, and α-CDT1 rabbit polyclonal antibodies (Cell Signaling Technologies), α-CUL2 rabbit polyclonal (Novus Biologicals), α-γH2AX mouse monoclonal (Upstate), α-UBE2M and α-Tubulin mouse monoclonal antibodies (Abcam), α-WEE1 and α-RAD51 rabbit polyclonal, and α-BRCA1 mouse monoclonal antibodies (SantaCruz Biotechnology).

### Immunofluorescent microscopy

**Figure 1:**
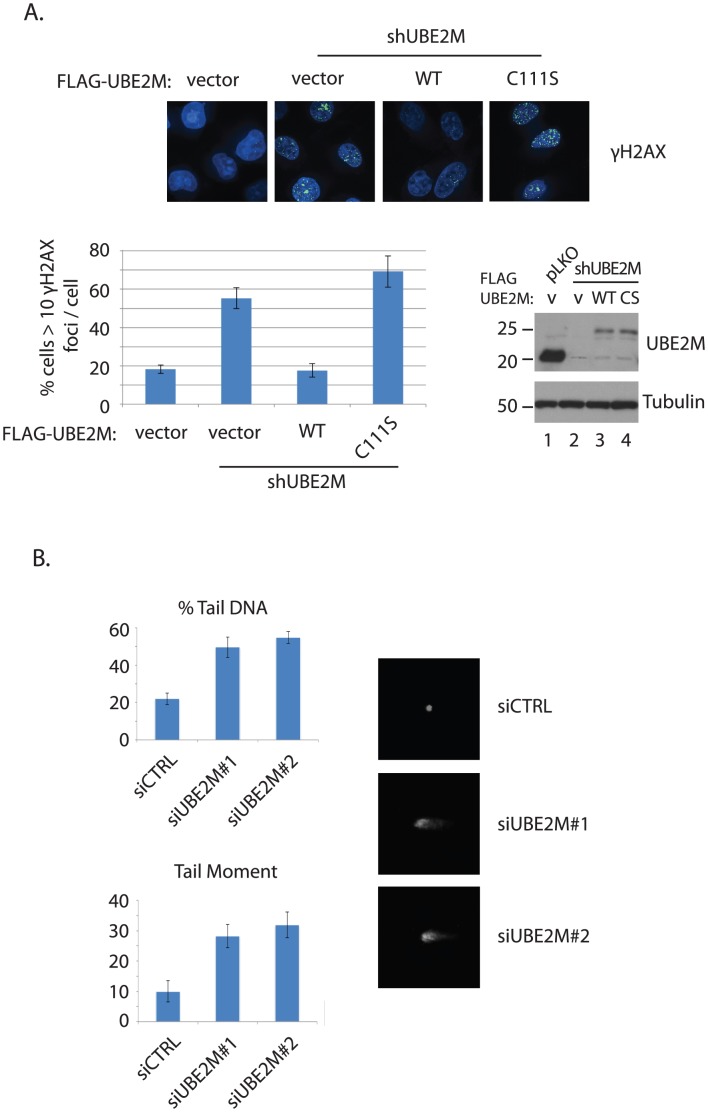
Disruption of genomic integrity upon UBE2M silencing. **A**. γ-H2AX foci was measured upon expression of shRNA targeting UBE2M 3′UTR, then rescued by expressing siRNA-resistant UBE2M WT or C111S mutant. The western blot analysis shows the knockdown efficiency and the expression of FLAG-HA tagged UBE2M WT and C11S mutant. (∼3 kDa shift is predicted). **B**. Neutral comet assay. HEY cells were transfected with control or two independent UBE2M siRNAs for ∼72 hours before harvest for the analysis. The tail moment is the length of the tail times the density of the tail. % tail DNA is the density of the tail divided by the density of the tail plus the density of the head.

48∼55 hours after siRNA transfection (or shRNA transduction), cells were washed and pre-extracted with 0.25% Triton X-100 for 3 minutes and then fixed with 4% paraformaldehyde for 10 min. For the rescue of UBE2M experiments in Figure 1A, HEY cells were infected with UBE2M shRNA targeting 3′UTR and 48 hrs post-infection cells were transfected with pOZ-FLAG-HA-UBE2M constructs. ∼48 hrs after the cells were washed, fixed, then pre-extracted with triton buffer. The fixed cells incubated with primary antibodies against γH2AX (Upstate) and RAD51 (Santa Cruz Biotechnology) at 1∶500, followed by incubation with Alexa Fluor 488-anti-mouse (for γH2AX) or anti-rabbit (for RAD51) (Invitrogen). Vectashield mounting medium for fluorescence with DAPI (Vector Laboratories Inc, CA) was used to stain cellular nuclei. Images were collected by a Zeiss Axiovert 200 microscope equipped with a Perkin Elmer ERS spinning disk confocal imager and a 63x/1.45NA oil objective using Volocity software (Perkin Elmer). We counted 70–120 cells from each sample for generating statistical figures for γH2AX and RAD51 foci.

### GFP-based DNA repair assays

The U2OS cell line expressing an integrated homologous recombination reporter DR-GFP has been described [43], and the U2OS cell line expressing the NHEJ reporter was obtained from Dr. Jeremy Stark [44]. For NHEJ, the reporter contains a promoter that is separated from a GFP coding cassette by a puromycin resistance gene that is flanked by two I-SceI sites. Once the puro gene is excised by NHEJ repair of the two I-SceI-induced DSBs, the promoter is joined to the rest of the expression cassette, leading to restoration of the GFP marker. 48 hrs post-I-Sce1 transfection, cells were harvested and analyzed via flow cytometry for recombination efficiency using a BD Accuri C6 flowcytometer (BD Biosciences, Franklin Lakes, New Jersey). C-Flow software was used to analyze percent GFP-positive cells relative to the total number of the transfected cells. Approximately 30,000 cells were counted from each sample.

### Cell cycle analysis

HEY cells were synchronized at the G1-S interface by a double-thymidine block. On the first day, the cells were plated and incubated 24 hrs at 37°C in antibiotic-free RPMI. Cells were then treated with 2 mM thymidine for 18 hrs, followed by washing and release into fresh media for 8 hours. Cells were then treated with 2 mM thymidine for 18 hrs and then released with two washouts of RPMI media. Cells were harvested at various times post-release (3∼9 hrs). To analyze the effects of siRNAs for synchronous cell cycle, the cells were transfected with siRNAs on the first day when the cells were plated. For studying the effects of MLN4924 on cell cycle using double thymidine block, the cells were treated with 0.3 µM MLN4924 for 6 hrs prior to the final release from thymidine. For studying the effects of expressing CUL2 mutant on cell cycle using double thymidine block, all the procedures were the same except that the plasmids were transfected during the first release period. Cells were trypsinized and resuspended in 0.5 mL PBS and washed in 0.5 mL of PBS twice and then fixed in 0.5 mL 70% ethanol and incubated at 4°C for 24 hrs. Cells were then washed twice with PBS and resupended in 0.44 mL of 50 µg/mL of propidium iodide in 1% Triton X-100 and 0.06 mL of 200 µg/µL RNAse was added and incubated at 22°C for 1 hr. Approximately 45000 cells were counted from each sample.

### Neutral Comet assays

Comet assays were conducted under neutral conditions to assess DNA double strand breakages following the Trevigen Neutral Comet Assay protocol. Briefly, Hey cells were transfected with siRNA (CUL1–4A, UBE2M, UBE2F) or control siRNA (All-Star Negative) for 72 hrs. After which, cells were harvested and coated onto slides. Cells were lysed in (Trevigen Lysis Solution) for 1 hr at 4°C. Cells were then subjected to electrophoresis at 13 V for 35 min (1 V/cm). Cells were stained for 30 minutes with 1∶10000X SYBR Gold (Life Technologies, Carlsbad, CA). 50∼100 cells were counted for generating statistical figures. The comet tail moment, % Tail DNA, and tail length were analyzed by Image J.

### Cellular growth analysis

Cells were transfected with siRNA (CDT2, UBE2M, CUL4A), control siRNA (All-Star Negative; Qiagene), or cells were infected with virus containing the UBE2M shRNA (pLKO). Varying concentrations of camptothecin or a PARP inhibitor ABT888 (Selleck chemicals). After 5∼7 days, the cells were stained with crystal violet, and dried colonies were dissolved and resuspended with Sorensen buffer, then the colorimetric intensity of each solution was quantified using Gen5 software on a Synergy 2 (BioTek, Winooksi, VT) plate reader.

## Results

### Disruption of genome integrity upon UBE2M silencing

In order to test whether UBE2M is required for genome integrity, we silenced UBE2M using shRNA and measured the accumulation of γ-H2AX, a marker for DNA double strand breaks (DSBs). Silencing of UBE2M significantly increased the cells with γ-H2AX foci (Figure 1). The phenotype is rescued by expressing the RNAi-resistant wild type UBE2M but not with the UBE2M mutant in which the catalytic cysteine is mutated (C111S [45]; Figure 1), consistent with the notion that UBE2M forms a nedd8-thioester formation which is required for the neddylation cascade. Consistent with the γ-H2AX foci formation, silencing of UBE2M significantly accumulated double strand breaks (DSBs) in the genome in the neutral comet assay compared to control, suggesting that the damaged DNA is left unrepaired in the UBE2M-depleted cells (Figure 1B). Knockdown of UBE2F, another E2 nedd8 conjugating enzyme, did not cause elevated DSB (Figure S1 in File S1), suggesting that UBE2M is indeed the main E2 neddylating enzyme responsible for maintaining genome integrity.

### UBE2M silencing disrupts DNA damage response

**Figure 2:**
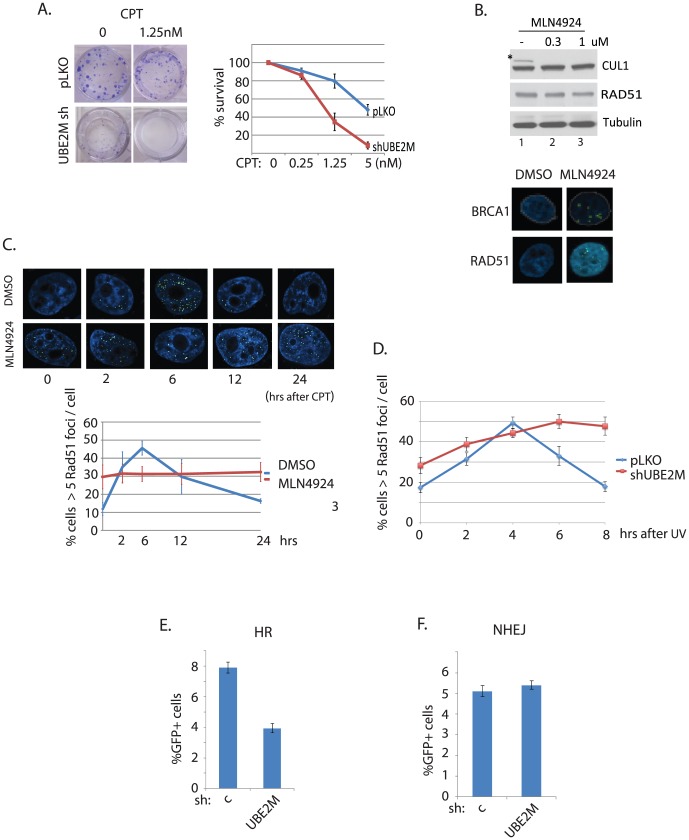
DNA damage response is perturbed by UBE2M silencing. A. Growth suppression by UBE2M silencing is enhanced by DNA damaging agents. Growth sensitivity of HEY cells in the presence of CPT and PARP inhibitor ABT888 (Figure S2 in File S1) was monitored using clonogenic assay. **B**. Treatment of HeLa cells with MLN4924 (0.3uM) leads to elevated BRCA1 and RAD51 foci formation. *indicates neddylated form. **C**. Time course study of RAD51 foci recruitment and resolution upon MLN4924 treatment. D. Time course study of RAD51 foci in UBE2M knockdown cells. **E**. Cells depleted of UBE2M were analyzed for HR (**E**) and NHEJ repair (F) assays.

We previously reported that treatment of cells with the neddylation inhibitor MLN4924 minimizes early DNA damage response in cultured cells [35], and a delay of early DNA damage response upon MLN4924 treatment was reported [33]. Therefore, we hypothesized that DNA damage response is perturbed upon inhibiting the protein neddylation, and this partially accounts for the disruption of genome integrity. RNAi-mediated silencing of UBE2M, the primary E2 Nedd8 conjugating enzyme, caused significant reduction in the proliferation of ovarian cancer HEY cell line (Figure 2A), consistent with the effects of MLN4924 on cell growth [31]. Treating the HEY cells with a Topoisomerase I inhibitor Camptothecin (CPT), further suppressed the cell survival of the UBE2M knockdown cells (Figure 2A), compared to control cells. Cells that are defective in DSB repair, particularly HR (homologous recombination) repair, are often hypersensitive to PARP inhibitors. Silencing of UBE2M caused the HEY cells to be more sensitive to a PARP inhibitor than control cells (Figure S2 in File S1). This suggests that UBEM knockdown may cause impairment of HR activity. These results suggest that UBE2M is required for cell survival, and that DNA damage response may be disrupted by UBE2M depletion.

HR repair-deficient cells often display defective RAD51 foci formation. Treatment of a pharmacological Neddylation inhibitor MLN4924 spontaneously increased RAD51 or BRCA1 foci formation (Figure 2B), consistent with a previous report that MLN4924 induces spontaneous DNA damage [31]. However, a delay in the rate of initial RAD51 foci formation was observed in the kinetic studies using MLN4924 (Figure 2C) and UBE2M knockdown cells (Figure 2D). Albeit the initial delay in the foci formation, the foci persisted without being resolved at later time points, compared to control cells. The aberrant RAD51 foci kinetics may potentially contribute to the PARPi sensitivity, and is further reflected by impaired HR reporter activity upon UBE2M knockdown (Figure 2E; two siRNAs shown in Figure S3 in File S1, and GFP expression control is shown in Figure S4 in File S1). The NHEJ reporter activity (Figure 2F) remained unaffected. Altogether, these results suggest that UBE2M and protein neddylation is required for maintaining genome integrity, and normal DNA damage response is impaired.

Inhibition of the proteasome function abrogates HR repair [39], [40], and impaired NFkB activation was suggested to be partly responsible for decreased expression of DNA repair genes such as RAD51 [46], [47]. Since a Cullin ligase mediates degradation of IkB and leads to NFkB activation [48], we tested whether the defects in HR repair is due to deficiency in the NFkB activation. Treatment of MLN4924 or knockdown of IkB did not significantly cause change in the expression level of RAD51 or FANCD2 (Figure S5 in File S1), suggesting that impaired NFkB function may not play a major role in the effect of UBE2M in DNA repair.

### Cullins mediate the effect of UBE2M on genome integrity

**Figure 3:**
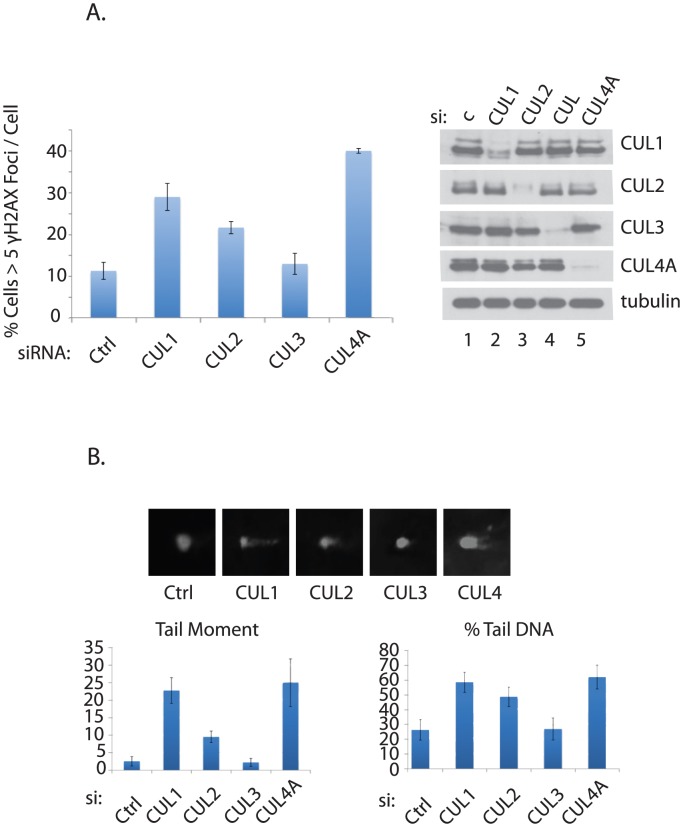
Effects of individual Cullin silencing in genome integrity. **A**. γ-H2AX foci induction was measured in HEY cells treated siRNAs against indicated cullins. Knockdown efficiency is shown in right. **B**. Formation of double strand breaks were measured using neutral comet assay, in HEY cells treated with siRNAs against indicated cullins.

UBE2M induces neddylation of multiple cullin E3 ligases, including Cullin (CUL) 1, 2, 3, and 4, thereby regulating their activities [1], [4]. Consistently, knockdown of UBE2M inhibited neddylation of CUL 1∼4 (Figure S6 in File S1). Therefore, we attempted to investigate to which extent inactivating each Cullin ligase impacts genome integrity. Knockdown of CUL1, CUL2, and CUL4 significantly induced H2AX foci, suggesting that these Cullin ligases are required for the genome integrity (Figure 3A). Consistently, the comet assay indicates that double strand DNA breaks are increased upon CUL1, CUL2, and CUL4, and to lesser extent upon CUL3 knockdown (Figure 3B). These results suggest that UBE2M inactivation may disrupt multiple aspects of DNA damage response through different Cullins.

### G1-S transition is impaired by silencing CUL2

**Figure 4:**
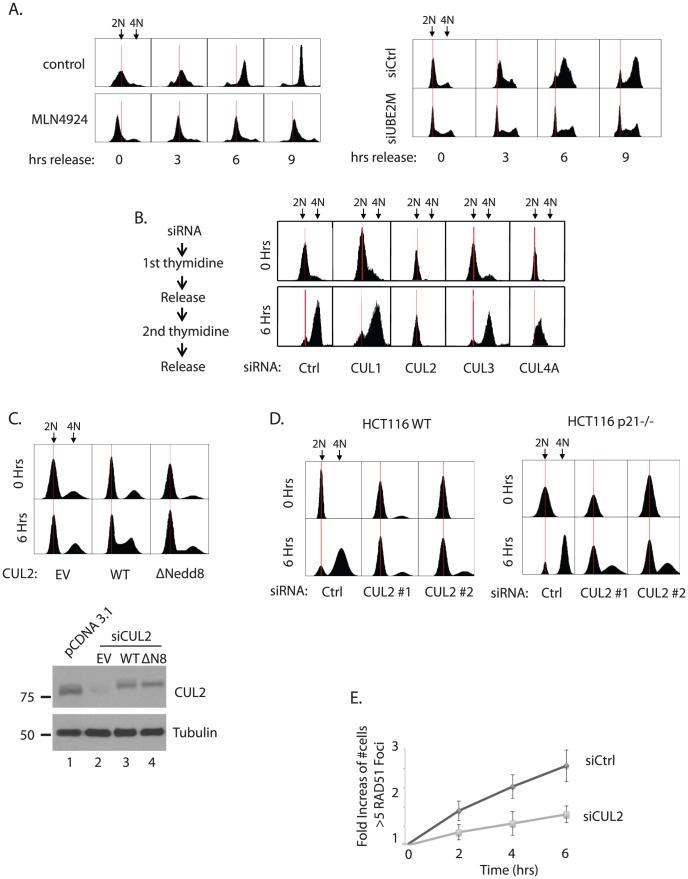
Inhibiting CUL2 neddylation leads to impaired G1-S transition. **A**. Double thymidine block experiments were performed in HEY cells treated with DMSO control or MLN4924 and UBE2M siRNA. See Experimental procedure for detailed protocol. The red line was established by selecting the peak value of the cells in G1 (2N) for the control siRNA sample at the zero hour time point. The red line was then kept constant between samples to provide a means of comparison. **B**. Double thymidine block experiments were performed in HEY cells individually knockdown with indicated cullins. **C**. Expression of siRNA-resistant CUL2 wild type (WT), but not the empty vector (EV) nor the CUL2 mutant (C689R; ΔNedd8 in the figure), partially rescues the G1-S arrest phenotype. CUL2 siRNA #3 targets the 3′UTR of the CUL2 mRNA. Western blot confirms the knockdown efficiency and ectopic expression of CUL2 proteins. **D**. Double thymidine block experiments were performed using the HCT116 wild type or p21-/- cells that are treated with either control or CUL2 siRNAs. **E**. Induction rate of RAD51 foci was measured in HEY cells treated with control or CUL2 siRNAs. The counting was *normalized* to the 0 time point to indicate the fold increase.

Among many other possible mechanisms for the delayed induction of RAD51 foci observed in Figure 2, we reasoned that perturbed cell cycle progression, specifically in the transition to S phase, can cause a disruption in the DNA damage dependent foci. Indeed, treatment of cells with MLN4924, or UBE2M silencing, significantly delayed or arrested the transition into S phase in the double thymidine block experiments (Figure 4A), suggesting that overall protein neddylation is required for the S phase entry. To systematically investigate to which extent inactivating each Cullin ligase impacts the G1 to S transition, we silenced individual Cullins and analyzed the G1-S transition. CUL1 is known to promote the G1-S transition in part via degradation of p27 [49], yet a significant delay of G1-S transition upon CUL1 knockdown was not observed in our assay condition (Figure 4B; the knockdown efficiency shown by western blots in figure 3). Silencing of CUL2 significantly impaired the S phase entry (two independent siRNAs are shown Figure S7 in File S1), while CUL4A knockdown had a milder delay. (CUL4B knockdown had a negligible effect; not shown). The G1-S arrest phenotype of CUL2 silencing was partially rescued by expression of wild type CUL2 but not by the CUL2 mutant that cannot be neddylated (K689R; [50]), underscoring the significance of neddylation in influencing the activity of CUL2 E3 ligase in promoting the cell cycle transition (Figure 4C). CUL2 is known to regulate the p21 stability to regulate the actin based cell motility, however this mechanism is not linked to cell cycle progression [51]. Consistent with this report, the ability of CUL2 to promote S phase entry is largely independent of p21 degradation, as silencing CUL2 led to the G1-S arrest in both wild type and p21-/- HCT116 cells (Figure 4D). To test whether the delayed G1-S transition due to the CUL2 inactivation is responsible for the delayed RAD51 foci formation, we analyzed the foci induction kinetics of RAD51. While CUL2 knockdown led to spontaneous induction of RAD51 foci to a milder degree, the rate of early induction of RAD51 foci is lower than that in control cells (Figure 4E). This result suggests that the delayed induction of RAD51 foci observed upon UBE2M silencing is, at least in part, due to the delay of the G1-S transition via CUL2 inactivation.

### Silencing CUL4-CDT2 leads to G2-M arrest and heightened RAD51 foci retention that is associated with DNA repair defects

**Figure 5:**
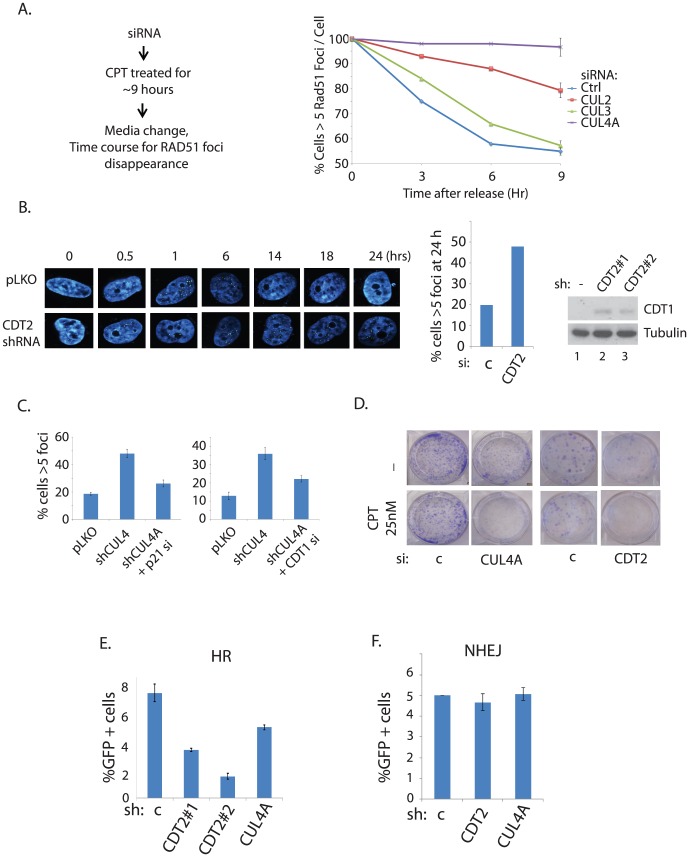
Silencing of CUL4 leads to G2-M checkpoint activation that is associated with DNA repair defects. **A**. Resolution of RAD51 foci was measured upon knockdown of individual Cullins. Schematic of the experiment shown in left. **B**. RAD51 foci kinetics was performed in cells in which CDT2 was stably knockdown. **C**. Prior depletion of CDT1 or p21 by siRNAs partially rescues the hype-RAD51 foci formation in MLN4924 treated cells. **D**. Clonogenic assays were performed for the HEY cells knockdown with CUL4A or CDT2. **E**. HR repair assays **F**. NHEJ assay.

Heightened G2-M checkpoint is associated with CUL4 knockdown cells [30], similar to the cells treated with MLN4924. We reasoned that the elevated G2-M checkpoint upon CUL4 knockdown is responsible for the persistent RAD51 foci observed in the UBE2M knockdown cells in Figure 2C. To test this, we analyzed the RAD51 foci resolution kinetics upon CPT treatment, in cells individually knockdown with each Cullin (Figure 5A). Cells were treated with low doses of CPT, followed by removal of the CPT media for indicated time points. In a condition where RAD51 foci is resolved by ∼9 hours, CUL4 knockdown cells maintained high level of RAD51 foci, while CUL2 or CUL3 knockdown cells lead to little or negligible degree of RAD51 foci retention, respectively, suggesting that CUL4 inactivation is mainly responsible for the elevated RAD51 foci formation observed upon UBE2M. Knockdown of CUL1 combined with CPT treatment led to significant cell death, thus was eliminated from our analysis. CUL4/CDT2-mediated degradation of CDT1, a replication origin licensing factor, was linked to the G2-M checkpoint control [28]. Consistently, silencing of CDT2 led to persistent RAD51 foci in the kinetic analysis, similar to that of UBE2M depleted cells (Figure 5B). The CUL4-CDT2 E3 ligase complex is known to regulate DNA re-replication and G2-M checkpoint through degradation of CDT1 and p21 [30], [52] Interestingly, depletion of CDT1 or p21 partially rescued the persistent RAD51 foci upon CUL4 knockdown (Figure 5C), suggesting that increase in DNA re-replication may be at least partially responsible for the persistent RAD51 foci. These results reaffirm that inactivation of the CUL4-CDT2 E3 ligase leads to heightened G2-M checkpoint that leads to persistent RAD51 foci formation. It is not clear whether the persistent RAD51 foci upon CUL4 or CDT2 silencing is indicative of the active HR repair, or is a reflection of HR repair being defective. For instance, a defect in downstream HR mechanism can result in persistent RAD51 foci. Silencing of CUL4 or CDT2 led to suppression of cell growth, which are further aggravated by CPT treatment (Figure 5D), suggesting that DNA repair mechanism may be disrupted. Consistently, HR repair activity is reduced upon CUL4 or CDT2 silencing (Figure 5E), coupled with the increased DSB formation observed in the comet assay (Figure 3A). Altogether, CUL4-CDT2 is required for promoting G2-M transition and genome integrity, and inactivation of these factors mainly accounts for the persistent RAD51 foci observed upon UBE2M silencing.

### DNA damage induced by UBE2M inactivation is partially dependent on CDT1 and p21

**Figure 6:**
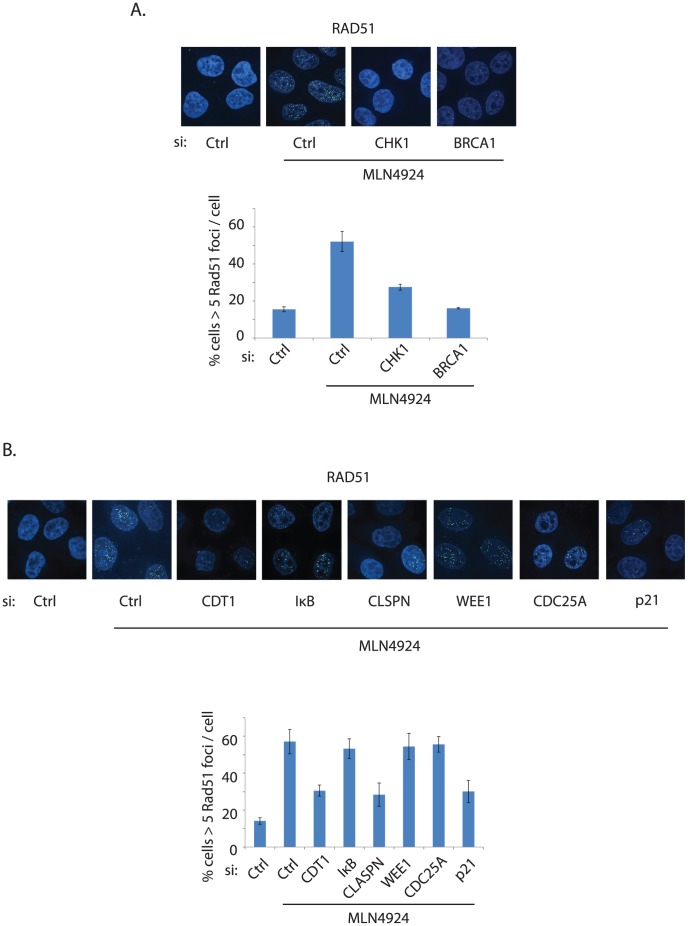
Depletion of CDT1, p21, and CLASPN partially rescues the hyper DNA damage response in MLN4924 treated cells. Knockdown efficiency is shown in Figure S8 in File S1. Knockdown of WEE1 was notably toxic, and only viable cells were counted.

To further investigate the relevant mechanisms of the Cullins that may attribute to the heightened DNA damage response phenotype observed in UBE2M-depleted cells, we interrogated several siRNAs against known substrates of Cullin ligases. We used MLN4924 treatment for convenience of experimental setup. siRNA treatment of CHK1 and BRCA1, known to be required for RAD51 foci induction, led to significant reduction of RAD51 foci induced upon MLN4924 treatment (Figure 6A), validating our approach that DNA checkpoint components are required for the RAD51 foci formation. Among a few known substrates of Cullin ligases that are implicated in cell cycle and genome integrity, silencing of CDT1 and p21 led to significant reduction of RAD51 foci induced by MLN4924 (Figure 6B; siRNA efficiency shown in Figure S8 in File S1). Knockdown of Claspin, a protein implicated in DNA checkpoint activation [53], also led to significant reduction of the RAD51 foci, further suggesting the requirement of checkpoint proteins in inducing a persistent DNA damage response. These results further underscore that deregulation of replication licensing may be a critical determinant in triggering a hyper DNA damage response and perhaps disruption in genome integrity, when protein neddylation is disrupted.

## Discussion

Our results suggest that inactivating the UBE2M E2 neddylating enzyme strongly disrupts genome integrity, through multiple aspects of the DNA damage response. Growth suppressive effects of cells by silencing UBE2M is further aggravated by DNA damaging agents, implying that disruption in the DNA damage response contributes to the cellular sensitization. A previous report showed that UBE2M and RNF111 RING E3 ligase induce neddylation of histone H4, which leads to activation of downstream DNA damage responses through 53BP1 and BRCA1 [2]. This mechanism could at least partly explain the delayed induction of RAD51 foci and reduced HR repair upon UBE2M silencing (Figure 2). In addition to the function through RNF111, our analysis of individual Cullin ligases further revealed that the impaired G1 to S transition, primarily incurred via defective neddylation of CUL2, contributes to the impaired entry to S phase and thus delayed induction of RAD51 foci. Albeit to the delayed induction of DNA damage response, the RAD51 foci remain persistently high without being resolved upon UBE2M depletion, coupled with accumulation of double strand breaks and impaired HR activity (Figure 2). These observations may suggest that the DNA damage incurred upon UBE2M depletion is not properly repaired, and further suggests that DNA repair activity is compromised in the UBE2M depleted cells. Among the UBE2M-interacting Cullins, CUL4A depletion noticeably increased the persistent RAD51 foci that were not resolved with treatment of DNA damaging agents (Figure 5A) that were coupled with accumulated DNA damage. The CUL4-CDT2 ligase has been linked to regulation of the dNTP pool, via degradation of an inhibitor of ribonucleotide reductase in *S. pombe* [54]. Thus, CDT2 knockout cells cannot repair double strand breaks properly, as the HR repair requires DNA synthesis and dNTPs. Our observation that CDT2 knockdown in HeLa cells sensitizes to CPT (Figure 5D) is consistent with this observation, however, whether a similar mechanism exists in mammalian cells in unknown. We also speculate that the small degree of DNA re-replication induced upon CUL4-CDT2 inactivation may deplete the dNTP pool, which may downregulate HR capacity. The CUL4-CDT2 may be more directly linked to HR repair, as CDT2-mediated degradation of anti-recombinogenic factor FBH1 can enhance HR repair activity [55]. Therefore, it is likely that multiple mechanisms can contribute to the persistent RAD51 foci observed in the CUL4 and UBE2M depleted cells.

**Figure 7:**
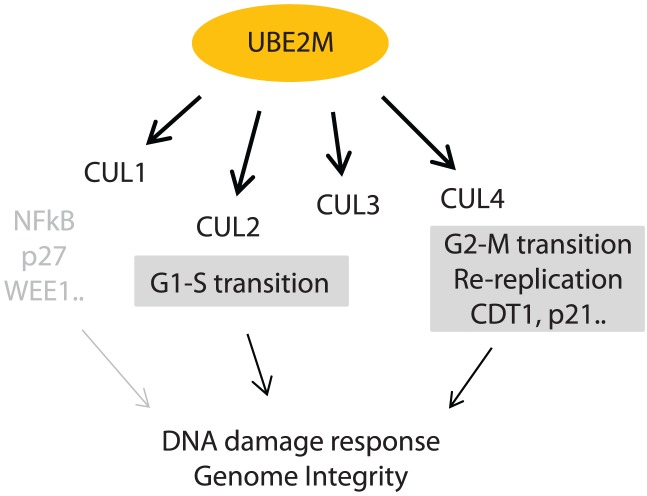
Model. UBE2M inhibition impacts DNA damage response and genome integrity involving multiple Cullin ligases.

Our data supports the idea that UBE2M could potentially be an alternative therapeutic target for increasing genome instability in cancer cells. Elevated expression of CUL1 and CUL4A is observed in cancers [56]–[59], and UBE2M expression is elevated upon irradiation in cancer cells [60], suggesting the UBE2M-Cullin components are required for survival of the cancer cells. RBX1, a RING E3 component of the Cullin complexes, is also required for maintaining genome integrity, by modulating the DNA replication licensing proteins (23). Our results that depleting CDT1 or p21 partially rescue the heightened DNA damage response of UBE2M-knockdown cells is consistent in that uncontrolled DNA re-replication may be a primary cause of such a phenotype (Figure 7). In addition to the well-known roles of CUL1 and CUL4-mediated regulation in DNA checkpoint and cell cycle progression, we further demonstrate that CUL2 knockdown impairs cell cycle progression and DNA damage response, leading to disruption in genome integrity. While we observed an elevated p21 level upon CUL2 knockdown as reported [51], the G1-S arrest phenotype of CUL2 depleted cells appear to be independent of p21 (Figure 4). The precise mechanism for the role of CUL2 ligase in promoting the G1-S transition remains to be determined.

In conclusion, the data demonstrate that the UBE2M E2 enzyme plays critical roles in maintaining genome integrity at least in part through neddylation of Cullins, and supports the notion that impairment of normal DNA damage response caused by inhibiting the overall protein neddylation can be exploited for designing combinatorial therapy for increasing chemosensitivity in cancer treatment.

## Supporting Information

File S1 Containing the following supporting information files: **Figure S1**. Comet assay. The tail moment is the length of the tail times the density of the tail. % tail DNA is the density of the tail divided by the density of the tail plus the density of the head. **Figure S2**. Growth suppression by UBE2M silencing is enhanced by PARP inhibitor ABT-888 in HEY cells. **Figure S3**. HR repair was was performed upon knockdown of UBE2M (two siRNAs) and CHK1. **Figure S4**. Expression level of GFP is unaffected by UBE2M knockdown. HeLa cells that stably express pLKO vector or pLKO-UBE2M shRNA were transiently transfected with pEGFP-N1 vector. 48 hours after, cells were harvested for flow cytometer analysis. **Figure S5**. MLN4924 or knockdown of IkB does not significantly affect the level of RAD51 or FANCD2 proteins. HeLa cells were simultaneously treated with siRNAs against IkB-α and IkB-β, then treated with MLN4924, for western blot analysis. **Figure S6**. Knockdown of UBE2M inhibits neddylation of Cullins. **Figure S7**. Knockdown of CUL2 arrests the cell cycle at the G1-S boundary. Left: confirmation of two independent siRNAs. Right: Double thymidine experiments were performed using the two siRNAs against CUL2 in HEY cells. **Figure S8**. Confirmation of knockdown for indicated siRNAs.
(EPS)
